# Overlapping transport and chaperone-binding functions within a bacterial twin-arginine signal peptide

**DOI:** 10.1111/j.1365-2958.2012.08005.x

**Published:** 2012-02-27

**Authors:** Sabine Grahl, Julien Maillard, Chris A E M Spronk, Geerten W Vuister, Frank Sargent

**Affiliations:** 1 College of Life Sciences, University of DundeeDow Street, Dundee DD1 5EH, Scotland, UK; 2 ENAC-IIE/Laboratoire de Biotechnologie Environnementale (LBE), Ecole Polytechnique Fédérale de Lausanne (EPFL)Station 6, CH-1015 Lausanne, Switzerland; 3 Spronk NMR ConsultancyPylimo Gatve 6, LT-01117 Vilnius, Lithuania; 4 Department of Biochemistry, University of LeicesterLancaster Road, Leicester LE1 9HN, England, UK

## Abstract

The twin-arginine translocation (Tat) pathway is a protein targeting system present in many prokaryotes. The physiological role of the Tat pathway is the transmembrane translocation of fully-folded proteins, which are targeted by N-terminal signal peptides bearing conserved SRRxFLK ‘twin-arginine’ amino acid motifs. In *Escherichia coli* the majority of Tat targeted proteins bind redox cofactors and it is important that only mature, cofactor-loaded precursors are presented for export. Cellular processes have been unearthed that sequence these events, for example the signal peptide of the periplasmic nitrate reductase (NapA) is bound by a cytoplasmic chaperone (NapD) that is thought to regulate assembly and export of the enzyme. In this work, genetic, biophysical and structural approaches were taken to dissect the interaction between NapD and the NapA signal peptide. A NapD binding epitope was identified towards the N-terminus of the signal peptide, which overlapped significantly with the twin-arginine targeting motif. NMR spectroscopy revealed that the signal peptide adopted a α-helical conformation when bound by NapD, and substitution of single residues within the NapA signal peptide was sufficient to disrupt the interaction. This work provides an increased level of understanding of signal peptide function on the bacterial Tat pathway.

## Introduction

Protein targeting is an important activity in all cellular systems. The model bacterium *Escherichia coli* targets proteins to and across the cytoplasmic (inner) membrane by one of two mechanisms: the general secretory (Sec) pathway or the twin-arginine translocation (Tat) pathway (Natale *et al*., 2008). Substrates of both systems are synthesized as precursors with N-terminal signal peptides; however, signal peptides of the Tat pathway bear a highly conserved S-R-R-x-F-L-K ‘twin-arginine’ amino acid motif (Berks, 1996). Moreover, the Tat pathway differs from the Sec pathway in that it is responsible for the transmembrane translocation of fully-folded proteins (Berks *et al*., 2003). This is achieved by a unique membrane-bound Tat translocase that couples the transmembrane electrochemical gradient to protein transport. In *E. coli* the Tat translocase is thought to comprise a ‘signal recognition module’ made up of the TatB and TatC proteins (Tarry *et al*., 2009) and a protein-conducting channel formed by multiple copies of TatA/E-type proteins (Leake *et al*., 2008).

Most of the 27 Tat-targeted proteins in *E. coli* are cofactor-containing redox proteins (Sargent, 2007). Such proteins face additional biosynthetic challenges before the ultimate export step. In particular, they must ensure correct binding of their respective redox cofactors before transport is attempted. There are two ways in which the cell prevents premature export of immature, or unfolded, Tat substrates. First, the Tat translocase displays an intrinsic ‘quality control’ activity where it appears to actively abort export of unfolded proteins (DeLisa *et al*., 2003). Second, some cofactor-containing Tat substrates undergo a lower tier of quality control involving Tat signal peptide binding proteins – and this process has been coined ‘Tat proofreading’ (Maillard *et al*., 2007; Sargent, 2007).

The *E. coli* periplasmic nitrate reductase is encoded by the *napFDAGHBC* operon (Grove *et al*., 1996). The *napA* gene encodes the 90 kDa catalytic subunit that binds a [4Fe-4S] cluster and *bis*-molybdopterin guanine dinucleotide as cofactors (Jepson *et al*., 2007). NapA is synthesized as a precursor with an N-terminal Tat signal peptide that is removed following transport to the periplasm (Thomas *et al*., 1999). This type of nitrate reductase is widespread in the prokaryotic world, where it plays a central role both in the global nitrogen cycle and in the virulence of some pathogenic bacteria (Potter *et al*., 2001). The *napD* gene product is essential for nitrate reductase activity (Potter and Cole, 1999; Maillard *et al*., 2007), while the other products of the *nap* operon are either involved in electron transfer from quinol to NapA [e.g. *napGHBC* (Brondijk *et al*., 2004)], or in cofactor loading into and/or activation of NapA [e.g. *napF* (Olmo-Mira *et al*., 2004)].

Before export the signal peptide of NapA is bound tightly by NapD, a cytoplasmic Tat proofreading chaperone, and this binding is thought to shield the signal peptide from the translocase until cofactor loading is complete (Maillard *et al*., 2007). The structure of NapD has been solved using nuclear magnetic resonance (NMR) methods and it has been shown to be a small monomeric protein (87 amino acids) that adopts a ferredoxin-type fold; that is a single β-sheet comprising four antiparallel β-strands packed against two α-helices (Maillard *et al*., 2007). Initial experiments suggested the signal peptide binding site was located within the β-sheet face of NapD (Maillard *et al*., 2007).

In this work, the molecular basis of the interaction between NapD and the NapA Tat signal peptide has been investigated. A combination of genetic and biophysical approaches is used to establish that the NapD binding epitope on NapA is located towards the N-terminus of the signal peptide and partially overlaps with the twin-arginine motif. NMR methods demonstrate the signal peptide adopts an α-helical conformation in the NapD-bound state and reveals the molecular basis of signal peptide recognition by NapD. Substitution of a single amino acid within the NapA signal peptide (A17) by either glutamine or leucine is shown to be sufficient to abolish the interaction with NapD both *in vivo* and *in vitro*, and suppression genetics identifies an I19F single variant, and an A14T/A71T double variant, of NapD that can rebind the NapA A17Q signal peptide. This work provides fresh insight into chaperone-signal peptide interactions on the bacterial Tat pathway.

## Results

### Identification of the NapD binding epitope within the NapA signal peptide

During biosynthesis of the *E. coli* periplasmic nitrate reductase the NapA signal peptide (NapAsp) is the primary point of recognition for the NapD Tat proofreading chaperone (Maillard *et al*., 2007). The first aim of this work was to identify the key amino acid residues that constitute the binding epitope on NapAsp recognized by the NapD protein. Initially, a genetic approach was taken in which a bacterial two-hybrid (BTH) system was employed. The BTH system relies on the reconstitution of adenylate cyclase activity in an *E. coli cya* mutant using complementary fragments (‘T25’ and ‘T18’) of an adenylate cyclase domain from a secreted toxin of *Bordetella pertussis* (Karimova *et al*., 1998). Positively interacting partners can be readily identified on MacConkey-Maltose indicator plates, while the degree of interaction can be quantified using β-galactosidase assays. This method has proven powerful in characterizing Tat signal peptide/chaperone interactions (Maillard *et al*., 2007; Buchanan *et al*., 2008).

**Fig. 1:**
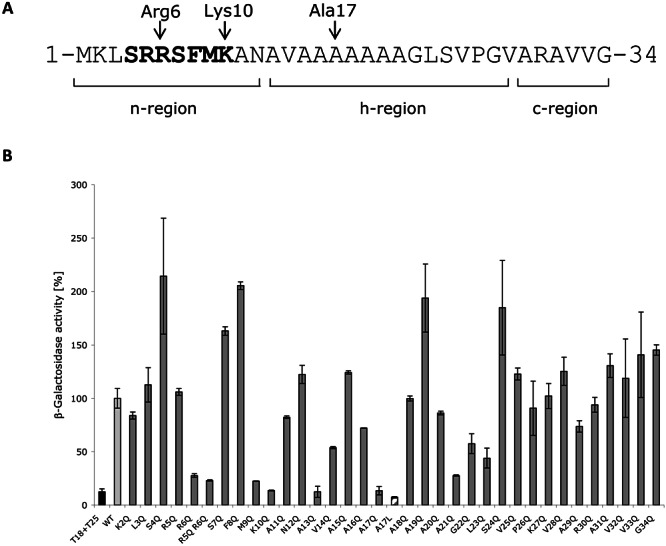
Mutagenesis of the NapA signal peptide. A. Amino acid sequence of the NapA twin-arginine signal peptide. The Tat motif is in bold and the N-terminal (n-), hydrophobic (h-) and C-terminal (c-) regions are indicated. Individual glutamine substitutions were introduced residue 2 through 34. The positions of R6, K10 and A17 are indicated by the arrows. B. Interactions between NapD and amino acid-substituted NapA signal peptides measured *in vivo* using a bacterial two-hybrid system. Vectors pT25-NapD and variants, encoding NapA–T18 fusions, were co-transformed into BTH101 and β-galactosidase activity determined. Activities are shown relative to that measured for the interaction between the native NapA signal peptide and native NapD (‘WT’). The column marked ‘T18+T25’ is the negative control containing ‘empty’ vectors. Error bars represent standard deviation of the mean, *n* = 3–9.

A plasmid encoding a NapAsp-T18 fusion was coexpressed in an *E. coli* reporter strain with plasmid pT25-NapD (Fig. 1B) and an *in vivo* interaction between NapAsp and NapD was readily detected (Fig. 1B). Next, the NapAsp-T18 plasmid was mutagenized such that each *napA* codon from positions 2–34 (Fig. 1A) would encode glutamine [chosen as a polar amino acid only rarely found in Tat signal peptides (Cristobal *et al*., 1999)]. This bank of *napA* mutants was then screened against the vector encoding a T25-NapD fusion and the level of *in vivo* interactions scored (Fig. 1B). The data clearly show that substitution of NapA residues R6, M9, K10, A13 and A17 with glutamine result in very low levels of β-galactosidase activity, indicative of impaired recognition of these peptides by NapD (Fig. 1B). A second substitution of A17 with leucine corroborated these initial findings and was also observed to impair the interaction with NapD (Fig. 1B). Note also that residues R6, M9 and K10 all fall within the canonical twin-arginine motif required for protein transport, and that a double R5Q-R6Q variant signal peptide was also found to be impaired in NapD binding (Fig. 1B).

**Fig. 2:**
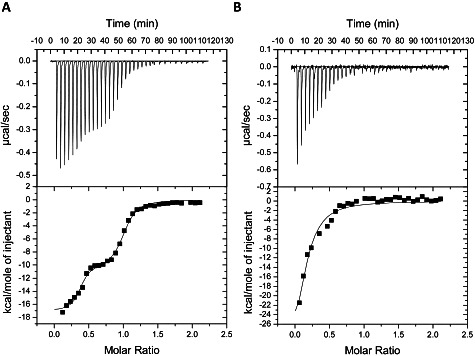
Microcalorimetric analysis of NapAsp binding by NapD. A. Calorimetric titration of 10 µM ϕMalE-NapAsp*^His^* by 100 µM NapD*^His^*. B. Calorimetric titration of 10 µM of the ϕMalE-NapAsp*^His^* A17Q variant with 100 µM NapD*^His^*. Titration conditions are listed in *Experimental procedures* and calculated thermodynamic parameters are listed in Table 1. Upper panels show raw data for heat effect during titration, lower panels are the binding isotherms.

In order to validate the *in vivo* two-hybrid results, isothermal titration calorimetry (ITC) was used as a sensitive *in vitro* protein–protein interaction technique. In order to allow the facile isolation of an intact NapA signal peptide a chimera was designed comprising maltose binding protein (MalE) linked via its C-terminus to a His-tagged NapA signal peptide (ϕMalE-NapAsp). Originally, Maillard *et al*. (2007) performed a titration with 50 µM NapD against 5 µM ϕMalE-NapAsp and recorded an apparent single binding event with a *K*_d_ ∼ 7 nM and an ‘*N*’ value (binding stoichiometry) of 0.59. Here, the experimental conditions were changed slightly such that 100 µM NapD was titrated against 10 µM ϕMalE-NapAsp (Fig. 2A). In this case, the binding curve became noticeably biphasic (Fig. 2A) corresponding to two distinct binding events – the first with a *K*_d_ ∼ 3 nM and the second with apparent *K*_d_ ∼ 143 nM (Table 1). Analysis of the binding stoichiometries (‘*N*’) points to a sum total of the molar ratios that is very close to 1.0 (Table 1). This suggests that, rather than two binding sites residing on one chaperone protein, these binding events involve two separate pools of protein each with a single binding site, but with 35% of the population binding with an apparent *K*_d_ of ∼ 3 nM and 64% of the population having a *K*_d_ of ∼ 143 nM (Table 1).

**Table 1:** Calorimetric analysis of NapA signal peptide binding by NapD

NapD	NapAsp variant	ITC data analysis	*N* value	*K*_d_ (nM)
Native	native	Two sites	Population 1 0.35 ± 0.02	3 ± 1
			Population 2 0.64 ± 0.02	143 ± 29
Native	R6Q	One site	Population 1 0.60 ± 0.02	725 ± 90
			Population 2 est. 0.40^a^	∞^b^
Native	M9Q	Two sites	Population 1 0.13 ± 0.01	1 ± 0.3
			Population 2 0.65 ± 0.01	75 ± 1
Native	K10Q	One site	Population 1 0.33 ± 0.05	1297 ± 498
			Population 2 est. 0.67^a^	∞^b^
Native	A17Q	One site	Population 1 0.16 ± 0.03	1100 ± 193
			Population 2 est. 0.84^a^	∞^b^

a Estimated *N*-value of putative second population.

b Binding constant outside measurable range.

Next, four of the NapA amino acid substitutions found to impair NapD binding *in vivo* (R6Q, M9Q, K10Q and A17Q) were introduced into the ϕMalE-NapAsp fusion protein. The interactions of each with NapD were then separately analysed by ITC (Table 1). NapD was observed to bind the M9Q version of the NapA signal peptide *in vitro* with similar characteristics to the native peptide (Table 1). It was concluded, therefore, that the *in vivo* result for M9Q (Fig. 1B) was a ‘false negative’, possibly resulting from instability of the NapA M9Q construct.

The remaining variant NapA signal peptides (R6Q, K10Q and A17Q) were all adversely affected in NapD binding compared with the native signal peptide (Table 1). In all three cases only a single binding event could be fitted to the data and the apparent *K*_d_s had increased by an order of magnitude to ∼ 1 µM (Table 1). In all three cases the binding stoichiometries remained well below 1.0 (Table 1), suggesting again that there are at least two populations of interactions at play with the majority of NapD molecules either not interacting at all with the variant signal peptides, or with binding that is too weak or transient to be detected by this technique. Taken together, the *in vivo* and *in vitro* interaction experiments point to NapA residues R6, K10 and A17 being directly involved in the NapD binding event, and together form an epitope for NapD binding towards the N-terminus of the twin-arginine signal peptide.

### The role of the NapA signal peptide in protein transport and nitrate reductase activity

To test the importance of the NapD binding epitope (NapA R6, K10 and A17) in the assembly and activity of the native nitrate reductase enzyme, three new strains were constructed. The LCB2048 parental strain (which produces only one active nitrate reductase, NapA) was genetically modified such that the *napA* R6, K10 and A17 codons were individually replaced with glutamine codons at the native *nap* locus on the *E. coli* chromosome. The new strains were designated SGQ061 (as LCB2048, *napA* R6Q), SGQ101 (as LCB2048, *napA* K10Q) and SGQ171 (as LCB2048, *napA* A17Q) respectively.

**Fig. 3:**
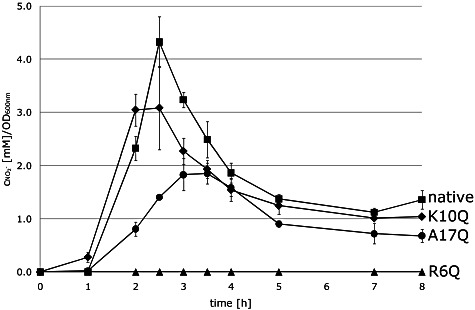
The role of NapA signal peptide residues in nitrate reductase activity. Strains carrying individual chromosomal point mutations in the *napA* gene were grown anaerobically in LB medium supplemented with 0.5% (v/v) glycerol and 0.4% fumarate (w/v) and 0.2% (w/v) nitrate. Nitrite production was recorded over time. Strains studied were: parent strain LCB2048 *napA*^+^ (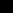), SGQ061 *napA* R6Q (▴), SGQ101 *napA* K10Q (◆), and SGQ171 *napA* A17Q (•).

The ability of SGQ061 (NapA R6Q), SGQ101 (NapA K10Q), and SGQ171 (NapA A17Q) to reduce nitrate to nitrite *in vivo* was assessed (Fig. 3). This assay reports on both the subcellular localization of NapA (only periplasmic enzyme can reduce nitrate in the living cell) and the levels of nitrate reductase activity in the periplasm. Under these assay conditions, the parental strain (*napA*^+^) showed a peak of nitrite production around 2.5 h following nitrate addition, which then gradually decreased to a stable, basal level, presumably as the nitrite was further reduced to ammonium by the periplasmic cytochrome *c* nitrite reductase. Conversely, the SGQ061 (NapA R6Q) strain did not display any detectible nitrite production even after 8 h incubation with exogenous nitrate (Fig. 3). Surprisingly, despite its apparent importance in NapD binding, the SGQ101 (NapA K10Q) strain retained periplasmic nitrate reductase activity close to that observed for the parental strain (Fig. 3). The SGQ171 (NapA A17Q) strain, however, displayed consistently lower levels of nitrate reduction than that detected for the parental strain (Fig. 3). In particular, the SGQ171 (NapA A17Q) strain produced nitrite relatively slowly, which peaked at a relative level less than half that observed for the parental strain (Fig. 3).

**Fig. 4:**
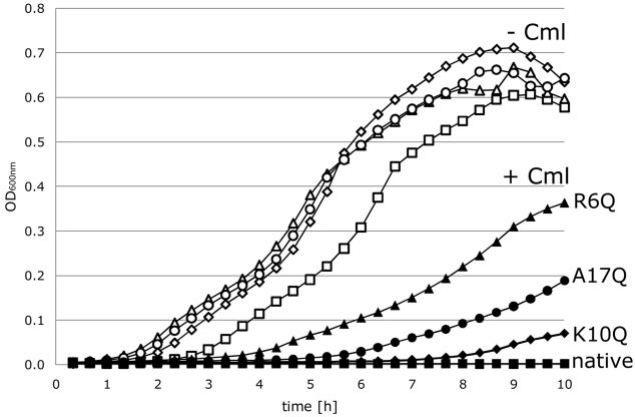
The role of NapA signal peptide residues in Tat transport activity. *In vivo* Tat transport assay. *E. coli* strain MG1655 (*tat^+^*) was transformed with derivatives of pUNI-NapA, which produce fusions between versions of the NapA signal peptide and the CAT enzyme (ΦNapAsp-CAT). Cells were grown aerobically in LB medium supplemented with 0.5% glycerol in presence (+Cml; closed symbols) or absence (−Cml; open symbols) of chloramphenicol (200 µg ml^−1^ final concentration). Constructs studied were: native NapA signal (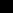), R6Q signal (▴), K10Q signal (◆), and A17Q signal (•).

To dissect these data into specific defects in either Tat transport or enzyme biosynthesis, signal peptide function was assayed in isolation. A fusion of the NapA signal peptide alone (NapAsp) to chloramphenicol acetyl transferase (CAT) was previously observed to efficiently transport the passenger protein to the periplasm, and so confer sensitivity to exogenous chloramphenicol as CAT is inactive in that compartment (Maillard *et al*., 2007). Here, this growth test was used to assess the Tat transport efficiency of the NapA R6Q, K10Q and A17Q variant signal peptides (Fig. 4). An *E. coli* strain (MG1655, *tat*^+^) was separately transformed with plasmids encoding ϕNapAsp-CAT and the NapAsp R6Q, K10Q and A17Q derivatives. Aerobic growth was then tested in the presence and absence of exogenous chloramphenicol (Fig. 4). The strain producing the fusion bearing the native NapA signal peptide was incapable of growth in the presence of chloramphenicol (Fig. 4), indicative of efficient targeting of the enzyme to the periplasm. Conversely, the strain producing the R6Q derivative of the NapAsp-CAT fusion was observed to be resistant to the addition of exogenous chloramphenicol at 200 µg ml^−1^, an indication of a defect in Tat transport activity (Fig. 4). Taken together, these data (Figs 3 and 4) are consistent with the NapAsp R6Q derivative being blocked in Tat transport activity (no physiological periplasmic nitrate reductase activity and impaired translocation of the CAT reporter protein).

Consistent with the data from nitrate reduction experiments (Fig. 3), the NapA K10Q variant signal peptide displayed only a minor defect in Tat transport activity when fused to the CAT reporter protein (Fig. 4). This suggests that both cofactor loading and protein targeting are not compromised by this signal peptide sequence change. The NapA A17Q variant signal peptide displayed an intermediate phenotype in the Tat transport assay (Fig. 4), which is indicative of a Tat transport defect in this variant signal peptide.

### The NapA signal peptide adopts an α-helical conformation upon binding NapD

**Fig. 5:**
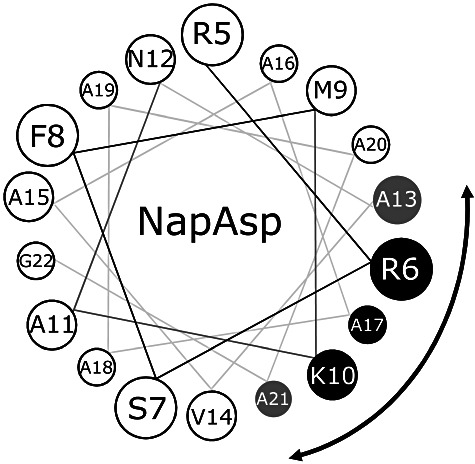
The NapD-bound NapA signal peptide adopts an α-helical conformation. Helical wheel projection of the NapA signal peptide showing, for clarity, residues R5 through G22. The residues making up the binding epitope for NapD are shown in black (for those confirmed by both *in vivo* and *in vitro* binding experiments) or grey (for those implicated by *in vivo* two-hybrid analysis only).

If the primary sequence of the NapA signal peptide is plotted as a canonical α-helix (3.6 residues per turn), then the R6, K10 and A17 residues all cluster together on a distinct face of the helix (Fig. 5). Moreover, if the α-helical model (Fig. 5) is correct this suggests that NapA A13 and A21 should also be close to the binding interface. Indeed, examination of the *in vivo* BTH data shows that both the A13Q and A21Q NapAsp variants are compromised for NapD binding *in vivo* (Fig. 1B). This analysis gives an initial indication that the NapA signal peptide may adopt an α-helical conformation during the NapD binding event; however, the structure of the NapA signal peptide, either before or after NapD binding, has not been reported. In order to address this, NMR-based approaches were taken.

The solution structure of ‘free’ NapD was solved by Maillard *et al*. (2007) using high resolution, multidimensional NMR techniques. However, previous NMR analysis of peptide binding involving titration of ^15^N-labelled NapD with a ϕMalE-NapAsp fusion (a 50 kDa polypeptide) led to a slow tumbling complex that resulted in line broadening (Maillard *et al*., 2007). In order to circumvent this problem here the NapA signal peptide (NapA residues M1 – V33) was covalently linked (via a short linker sequence – RSNLGIEGRPG) to the extreme C-terminus of NapD. The resultant NapDAsp chimera was stable to purification and ITC experiments showed that NapDAsp was unable to bind exogenously added NapA signal peptide (data not shown), which suggests strongly that the covalently linked signal peptide is bound within, and so is occluding, its native binding site on NapD.

Next, the NapDAsp fusion protein was produced in a form that was double-labelled with ^15^N and ^13^C. An overlay of the ^15^N-HSQC spectra of the NapD and NapDAsp proteins showed additional peaks in the latter originating from the additional residues of NapDAsp (Fig. S1). In addition, considerable differences in the positions of the cross-peaks previously assigned to native NapD were observed in the NapDAsp spectrum (Fig. S1). Together, these effects are indicative of the formation of a complex between the NapD core and the final ∼ 40 residues of the NapDAsp construct, which correspond to the NapA Tat signal peptide. Using triple-resonance NMR spectroscopy, the backbone and β-carbon assignments for residues T3-E85 of the NapDAsp protein (corresponding to the NapD part) and residues A109-A127 (corresponding to NapA signal peptide residues A11-A29) were made. Residues 86–108 of the NapDAsp fusion, which include NapA signal peptide residues M1-K10, could not be identified in the spectra, presumably because they are in intermediate exchange leading to line broadening beyond detection.

**Fig. 6:**
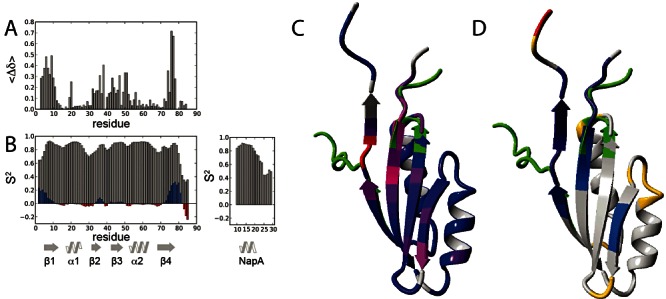
NMR analysis of the NapDAsp complex. A. Weighted, absolute chemical shift differences between NapDAsp and NapD as function of residue number. B. Chemical shift derived S^2^ order parameters (grey) as function of residue number. Differences with NapD are plotted as blue bars for increased S^2^ values indicating more order, and red bars for decreased values, indicating less ordered structure. Secondary structural elements are indicated. C and D. Ribbon representation of the superposition of NapD in ligand free form (PDB code 2jsx, first conformer) and peptide bound form (this work, see also Fig. 7). NapD regions with large conformational changes are coloured green. [C, corresponding to values of panel (A)] Ribbon colour coded blue to red for increasing chemical shift perturbation effects. [D, corresponding to data in panel (B)] Ribbon colour coded blue to violet for increased S^2^ values and yellow-orange-red for decreased values.

Figure 6A shows the weighted per-residue chemical shift differences for the NapDAsp fusion protein when compared with the free form of NapD. Upon signal peptide binding, the largest effects are observed for NapD residues T3-S9 in β-strand 1, D38 at the C-terminal end of β-strand 2, and L74-Q78 in β-strand 4, whereas residues of β-strands 2 and 3 with outward-facing side-chains also typically show significant effects (Fig. 6C). The measured chemical shifts were then used to predict both secondary structural elements, as well as S^2^ order parameters, which report on structural order of each individual residue (Fig. 6B). S^2^ values can range from 0, denoting complete disorder, to 1 for fully ordered, rigid structure, with values > 0.7 typical for regularly folded protein (Fig. 6B). Compared with the free NapD, the S^2^ values of NapDAsp show significantly higher values for the N-terminal and C-terminal regions of NapD (residues 3–6 and 75–79), indicating a more structured region for these parts of the molecule upon signal peptide binding (Fig. 6D). TALOS+ secondary structure predictions indicated the formation of an extended conformation of NapD residues 74–79, in line with the increased order parameters. Other secondary structure elements remain unchanged and the TALOS+ predicted ϕ, Ψ dihedral angles are consistent with the conformation of the free NapD. Although located in the core of NapD, the chemical shift effects observed for I19 indicate this residue senses the presence of the NapA signal peptide.

NapDAsp fusion protein residues A109-V123, corresponding to NapA signal peptide residues A11-V25, are well ordered and clearly in an α-helical conformation, as judged from the TALOS+ and NOE data. The signal peptide helical side-chains are followed by three residues in a turn-like conformation. Notably, to accommodate this turn NapA signal peptide residue G22 adopts a positive ϕ angle. Subsequent residues rapidly become more disordered, as judged from their S^2^ order parameter values.

**Fig. 7:**
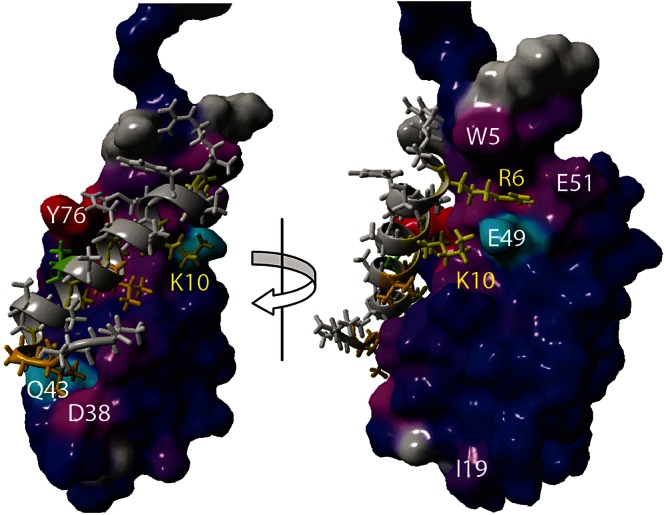
A Model for the NapD-NapA signal peptide complex. NapD is shown in surface representation, colour-coded blue to red for increasing chemical shift perturbation effects (as also shown in Fig. 6) and using white annotation for NapD residues. The NapA signal peptide is shown in ribbon and stick representation, using yellow colouring for crucial interacting residues and their annotation.

Next, chemical shift predicted structural data were used to generate a model for the complex (see *Experimental procedures*). Figure 7 shows a surface representation of the NapD protein, colour-coded according to the per-residue chemical shift pertubation, and the NapA signal peptide in stick representation. The signal peptide is positioned snugly on top of the β-sheet of NapD. The interface comprises an extensive surface with hydrophobic interactions and the close packing explains the need for the poly-Alanine character of the NapA signal peptide. The newly formed β-sheet residues of NapD, V75-Q79, line one side of the interaction interface and are paired with residues W5-V7 of β-strand 1 (Fig. 6C and D). Notably, V75 and Y76 together form a β-bulge. In the model, NapA signal peptide residue K10 engages in a salt–bridge interaction with E49 of NapD, with its aliphatic side-chain atoms packed against NapD V47. NapA signal peptide residue R6 wedges in between E49 and W5 of NapD, with its side-chain atoms packed against the W5 side-chain, whereas its guanadinium groups are oriented towards the highly acid β3-α2 loop-region of the NapD molecule comprising E51, D52 and E54. At the C-terminal end of the signal peptide, the backbone oxygen of NapA A21 engages in a hydrogen bond to the side-chain of NapD Q43, and the NapA L22 residue packs into a cavity formed by NapD residues V37 and the Hβ-atoms of D38. Notably, the Cβ chemical shift of D38 changes by > 3.0 p.p.m. upon signal peptide binding.

Overall, the NMR spectra and resultant structural model clearly show that the NapA signal peptide adopts a helical conformation upon NapD binding, and that the peptide binding site spans the length of the single β-sheet face of the NapD protein.

### Suppression genetics: isolation of NapD variants with affinity for NapAsp A17Q

Next, an attempt was made to genetically identify the regions of NapD important for the regulation of the interaction with NapAsp. This was tackled by searching for conformational suppressor mutations in *napD* that could restore binding to the variant signal peptides identified in this work (Fig. 5). To select for suppressors a genetic screen was employed based on the BTH system. The reporter strain BTH101 was co-transformed with a plasmid encoding either NapAsp R6Q-T18 or NapAsp K10Q-T18sp or NapA A17Q-T18 together with random libraries of *napD* mutants. These were prepared by error-prone PCR where the chosen theoretical error rates were 1.0% and 1.5%. The PCR products were cloned into vector pT25 resulting in around 100 000 clones per library. Suppressors (i.e. positive interactors) were detected on MacConkey maltose indicator plates where red colonies indicated an interaction.

The screen against NapAsp R6Q resulted in the isolation of 25 suppressors that could apparently restore interaction with this normally defective signal peptide. However, when the specificity of each newly isolated suppressor was tested by two-hybrid against the other glutamine-substituted NapA variants (K10Q and A17Q) it was clear that none of the 25 suppressors were specific for the R6Q substitution. It is possible that this screen has isolated variant NapD proteins with a generally enhanced affinity for the NapA signal peptide.

The screen against NapAsp M9Q resulted in no suppressors whatsoever, which is further evidence that the NapAsp M9Q-T18 fusion is unstable and a ‘false negative’ in this assay.

The screen against the NapAsp K10Q signal peptide identified three suppressors able to rebind this signal peptide. In this case, however, one of the suppressors was unable to interact with the R6Q or A17Q variants of the NapA signal peptide and so was considered specific for the K10Q substitution. Sequence analysis revealed four NapD amino acid substitutions were present in the product of this clone: S9G, I22T, V36I and G68S. This variant protein was not pursued further here.

Finally, the screen against the NapAsp A17Q signal peptide identified 12 suppressors apparently able to rebind this signal peptide *in vivo*. Here, three of the suppressors were highly specific for the A17Q substitution and sequencing of the mutant *napD* genes identified an I19F single variant, an A14T/A71T double variant, and an I19F/T59I double variant.

Overexpression vectors were constructed that would encode six different NapD variants: the I19F single substitution; the A14T/A71T and I19F/T59I double substitutions; and the A14T, T59I, and A71T single substitutions. Each variant was then purified and tested for signal peptide binding *in vitro* against native MalE-NapAsp and its A17Q variant by ITC (Table 2). As shown in Table 1, native NapD binds the native NapA signal peptide in a biphasic manner with a relatively low (3 nM) and a higher (143 nM) dissociation constant, while titration of native NapD against MalE-NapAsp A17Q resulted in a single binding event with an apparent *K*_D_ of 1.1 µM. In contrast here, when NapD I19F was titrated against MalE-NapAsp A17Q this resulted in a biphasic binding with a relatively low (14 nM) and a higher (704 nM) dissociation constant (*K*_D_) (Table 2, Fig. S2). These data suggest strongly that a single I19F substitution in NapD is sufficient to restore binding to the NapA A17Q signal peptide.

**Table 2:** Restored binding to the NapA A17Q signal peptide by NapD variants

NapD variant	NapAsp variant	ITC data analysis	*N* value	*K*_d_ (nM)
I19F	A17Q	Two sites	Population 1 0.29 ± 0.1	14 ± 6
			Population 2 0.62 ± 0.06	704 ± 312
I19F	Native	One site	0.97 ± 0.01	806 ± 71
I19F T59I	A17Q	Two sites	Population 1 0.25 ± 0.01	22 ± 8
			Population 2 0.65 ± 0.04	261 ± 107
I19F T59I	Native	One site	Population 1 0.39 ± 0.02	935 ± 142
			Population 2 est. 0.61^a^	∞^b^
T59I	A17Q	One site	Population 1 0.57 ± 0.01	633 ± 84
			Population 2 est. 0.43^a^	∞^b^
T59I	Native	One site	0.81 ± 0.01	328 ± 40
A14T A71T	A17Q	Two sites	Population 1 0.30 ± 0.01	12 ± 2
			Population 2 0.69 ± 0.01	218 ± 28
A14T A71T	Native	One site	0.91 ± 0.01	571 ± 26
A14T	A17Q	One site	0.80 ± 0.02	1,869 ± 216
A14T	Native	One site	Population 1 0.70 ± 0.01	763 ± 92
			Population 2 est. 0.30^a^	∞^b^
A71T	A17Q	One site	1.11 ± 0.01	833 ± 60
A71T	Native	One site	0.83 ± 0.01	313 ± 25

a Estimated *N*-value of putative second population.

b Binding constant outside measurable range.

The NapD A14T/A71T suppressor was also isolated and titrated against MalE-NapA_SP_ A17Q in the calorimeter (Table 2, Fig. S2). This also resulted in a biphasic binding curve (Fig. S2) with a relatively low (12 nM) and a higher (218 nM) dissociation constant (*K*_D_) (Table 2), which is broadly similar to that seen for the native interaction (Table 1, Fig. 2). As there were two amino acid substitutions in this suppressor the corresponding single substitutions were also analysed. Interestingly, the NapD A14T variant was essentially incapable of binding to the native NapA signal peptide, or the NapA A17Q variant (Table 2): when titrated against MalE-NapA_SP_ A17Q a single binding event was observed with an apparent *K*_D_ of ∼ 2 µM (Table 2). Similarly, the single A71T NapD variant bound MalE-NapA_SP_ A17Q with single binding event and an apparent *K*_D_ of 833 nM (Table 2). Thus, it appears that a combination of both A14T and A71T is necessary to restore native-like binding of NapD to the A17Q variant signal peptide.

Finally, the NapD I19F/T59I suppressor was isolated and titrated against MalE-NapA_SP_ A17Q (Table 2, Fig. S2). This too resulted in a biphasic binding curve (Fig. S2) with a relatively low (22 nM) and a higher (261 nM) dissociation constant (*K*_D_) (Table 2), similar to that seen for that observed for both native interaction (Table 1) and the interaction of the I19F single substitution. In this case the T59I amino acid substitution was found to not contribute much to the binding event. The NapD T59I single variant was isolated and titrated against MalE-NapA_SP_ A17Q. In this case only a single binding event was observed with an apparent *K*_D_ of 633 nM (Table 2).

Taken together, these experiments have identified a NapD I19F single substitution, and an A14T/A71T double substitution, as being sufficient to restore binding of NapD both *in vivo* and *in vitro* to the A17Q variant NapA signal peptide.

### Phenotypic characterization of NapAsp A17Q suppressors

Given that the introduction of the *napA* A17Q mutation onto the chromosome at the native *nap* locus resulted in a strain that had clearly reduced periplasmic nitrate reductase activity (Fig. 3), it was investigated here whether the new suppressors could restore full physiological NapA activity to the mutant strain. *E. coli* strain SGQ171 (as LCB2048, *napA* A17Q) was transformed with pT25-NapD-based plasmids encoding NapD I19F, A14T/A71T and I19F/T59I and the *in vivo* nitrite production assay carried out (Fig. S3). The SGQ171 (*napA* A17Q) strain characteristically produces nitrite after a long lag-phase of around 3 h, but none of the three identified suppressors were able to dramatically increase the degree of nitrate reduction exhibited by this strain (Fig. S3). Note, however, that strain SGQ171 still produces endogenous NapD. In order to address this, a new strain was constructed called LP203_S_ that, besides the chromosomal codon change for *napA* A17Q, also carries an in-frame deletion of *napD*. This strain (LP203_S_) was found to be completely devoid of any NapA activity (data not shown). Moreover, nitrate reductase activity could not be restored by any of the *napD* suppressor mutants either (data not shown). Clearly, then, the *napD* suppressor mutants can bind the NapA A17Q signal peptide both *in vivo* and *in vitro* but are apparently devoid of any physiological activity.

## Discussion

### Signal peptide recognition by the NapD chaperone

In this study NapA signal peptide residues R6, K10 and A17 residues have been identified as being central to the NapD recognition mechanism. When plotted as a canonical α-helical wheel, residues R6, K10 and A17 (as well as A13 and A21) are predicted to cluster together to form a distinct binding face on the signal peptide (Fig. 5). Sequence comparison of the Tat signal peptide from *E. coli* NapA with those of other bacteria shows that those residues of the NapD binding epitope are all very well conserved (Fig. S4), suggesting a conserved and important role for those residues in NapD binding in general.

The NapA R6 residue clearly has overlapping functions in both assembly and export of the nitrate reductase. A strain carrying a chromosomal NapA R6Q allele was completely devoid of nitrate reductase activity (Fig. 3), and a reporter assay suggested this substitution was a signal peptide defective in Tat transport (Fig. 4). These data clearly point to a role for NapA R6 in Tat transport. However, both *in vivo* and *in vitro* binding studies also identified R6 as being important for the NapD interaction (Fig. 1, Table 2). Taken together, this is suggestive that as well as the structural diversity observed in different Tat proofreading chaperones (e.g. the all-helical TorD family versus the ferredoxin-like NapD family) that there is also functional diversity in the different protein families. Indeed, initial studies of signal peptide binding by Tat proofreading chaperones focussed on the TorD family in general and the TorD-TorA system from *E. coli* in particular. The TorD binding epitope on the TorA signal peptide was mapped by glutamine-scanning mutagenesis using similar methodology as described here for NapD-NapA (Buchanan *et al*., 2008). However, in this case the TorD binding epitope was located at the C-terminus of the TorA signal peptide – relatively far, in molecular terms, from the conserved twin-arginine motif (Buchanan *et al*., 2008). Moreover, TorD family chaperones seem to be intimately linked with cofactor insertion and maturation of metalloenzymes, so much so that some non-exported enzymes rely on their activity for assembly (Ize *et al*., 2009). The NapD-NapA system is very different in this regard, as the NapA Tat motif at the N-terminus of the signal peptide overlaps with the NapD chaperone-binding epitope considerably (Fig. 5). This could suggest that NapD binding and Tat transport are co-evolved functions within the NapA signal peptide, and could further imply that the role of NapD is focussed entirely on the Tat transport step on nitrate reductase biosynthesis, rather than in cofactor insertion as is often assumed.

The NapA K10 residue lies within the Tat motif and is very highly conserved in nitrate reductase signal peptide primary sequences (Fig. S4). In this case, however, a K10Q substitution had little effect on overall the nitrate reductase activity *in vivo*. Similarly, a strain carrying a *napA* A17Q allele at the native chromosomal locus had low, but still detectible, periplasmic nitrate reductase activity. These data suggest cofactor insertion and protein export can continue to a significant extent in these mutants. This seems at odds, however, with the NapD binding experiments, which suggest both K10Q and A17Q NapA substitutions drastically impair NapD binding. Given that NapD is essential for nitrate reductase activity (Maillard *et al*., 2007), how can these results be reconciled? One possibility is that the detection limits of the BTH system, which these data suggest must lie above an apparent *K*_D_ of 750 nM (Fig. 1, Table 1), bears little relation to the binding constants required for NapD function in the living cell. Indeed, the variant NapA signal peptides reported here to be ‘devoid’ of chaperone binding still retain some ability to interact with NapD, with apparent *K*_D_s of ∼ 1 µM (Fig. 1, Table 1), and a binding constant of 1 µM could be considered relatively ‘tight’ for many other biological systems. It is therefore conceivable that NapD function is continuing to some extent in the mutant strains. The key experiment to test this, of course, was the deletion of the *napD* gene in the SGQ061, SGQ101 and SGQ171 mutant backgrounds. This resulted in all cellular nitrate reductase activity being lost (Fig. S3), which confirms that NapD can still functionally interact with the variant NapA signal peptides identified in this work.

Interestingly, ITC revealed a two-tier, biphasic binding event for the NapD interaction with the native NapA signal peptide (Fig. 2). This observation was apparently in contrast to previous published work (Maillard *et al*., 2007). At first glance, this biphasic binding curve may appear to suggest two binding sites for the signal peptide on each NapD protein; however, it should be noted that the total molar binding stoichiometry (*N*) consistently adds up to 1, as opposed to 2, and the subsequent NMR study described here revealed a single signal peptide binding site for NapD. Therefore, in this case such a biphasic binding curve could be best explained by either NapD or the NapA signal peptide existing as two subpopulations with subtly different properties. For example, similar biphasic binding between the *E. coli* NarJ protein and the N-terminus of NarG has recently been observed (Zakian *et al*., 2010). In addition, it was also demonstrated by NMR that the NarG N-terminus has only one binding site on NarJ (Zakian *et al*., 2010). In this case slight changes in pH were able produce single monophasic binding curves and it was concluded that NarJ consists in two subpopulations with different protonation states, and that this is sufficient to observe subtle differences in binding characteristics (Zakian *et al*., 2010).

### A model for the NapD–NapAsp complex

High-resolution NMR spectroscopy is a powerful technique for studying structure, dynamics and interactions of biomolecules. The NMR data presented in this study clearly indicate the formation of a specific complex between the tail of the NapDAsp fusion construct, corresponding to the NapA signal peptide, and the NapD protein (Figs 6 and 7). This interaction induces an extension of β-strand 4 of NapD but leaves the overall conformation of the NapD molecule unaffected. The signal peptide traverses the β-sheet region of NapD almost completely. NapA residues R6 and K10 are well-positioned to engage in ionic interactions with the negatively charged (and almost completely conserved) E49 and E51 residues of NapD. Indeed, a previously described E49A variant of NapD resulted in a 4.5 kCal mol^−1^ loss of binding enthalpy when titrated against the NapA signal peptide in an ITC experiment (Maillard *et al*., 2007), which can now be readily explained by a loss of the NapA K10/NapD E49 salt–bridge. Substitution of NapD E33, positioned just juxtaposed to E49, with alanine had little effect on the peptide binding enthalpy (Maillard *et al*., 2007). In line with this finding, NapA K10 faces away from NapD E33 with its amino group at > 6 Å distance from the NapD E33 carboxyl moiety. Moreover, a loss of 5.9 kCal mol^−1^ in peptide binding enthalpy upon substitution of the conserved NapD Q43 with alanine (Maillard *et al*., 2007) supports the observation made for the NapDAsp model in this work that Q43 is hydrogen bonded to the backbone oxygen of NapA A21 at the C-terminal end of the signal peptide helix. Indeed, the deleterious effects of further substituting alanine residues at the NapD–NapAsp interface by glutamine are now readily explained as this would immediately disrupt the tight packing observed in this region due to steric clashes.

During the course of this work, an alternative NMR structure of the NapD-NapAsp complex was deposited in the PDB (2pq4). As the experimental data pertaining to the 2pq4 structure have not been deposited in the public BMRB database, we could not fully verify our model against these data. However, our model agrees with the 2pq4 structure in the extension of NapD β-strand 4 and the positioning of NapAsp residues A21-L24 at the N-terminus of NapD β-strand 3. The major differences involve the helical nature of the signal peptide and its angle with respect to the NapD molecule (c.f. Fig. S5). Moreover, the 2pq4 structure does not provide a rational explanation for the NMR and interaction data presented in this work (c.f. Fig. S5): the importance of NapAsp R6 and K10 in NapD binding cannot be explained in the 2pq4 structure, nor can the prior *napD* mutagenesis data (Maillard *et al*., 2007). Indeed, the agreement between the chemical shift perturbation data and the 2pq4 structure is generally poor and the NapA signal peptide conformation in the alanine-rich region is in disagreement with the TALOS+ predicted dihedral angles. Assessment of the structural quality shows very poor overall scores for the 2pq4 complex (Table S1).

### Long-range effects on signal peptide recognition by NapD

Screening of a *napD* mutant suppressor library revealed the single substitution I19F, and the double variant A14T/A71T, as being able to restore binding to the NapAsp A17Q. NapD residue I19 is positioned at the N-terminal end of the first α-helix, and is therefore not located in direct vicinity of the NapD/NapA interface. However, a possible explanation for the effect of I19F on NapA_SP_ A17Q binding comes from inspection of our NapDAsp model. The NapD I19 side-chain forms hydrophobic interactions with L44 on β-strand 3 and the neighbouring residue, I45, has direct contact with NapAsp residue A17 in the complex. Thus, it is conceivable that a NapD I19F substitution could have a long-range influence on the conformation of I45, which could in turn allow binding of the NapAsp variant A17Q. In support of such an indirect mechanism are the chemical shift perturbations observed for NapD I19 and S20 (Fig. 6), which are the only residues on this face off the molecule that display significant effects upon signal peptide binding.

The effect of the A14T/A71T substitutions on NapD is more difficult to estimate because the NapDAsp model shows no direct binding of those two residues to the signal peptide. Clearly, only a detailed structural analysis of the NapD A14T/A71T – NapA A17Q complex will reveal the true molecular basis for this interaction.

### Concluding remarks

How does the NapDAsp model relate to other Tat chaperone-signal peptide pairs? There are no structures of TorD family chaperones in complex with peptides; however, mutagenic studies (Chan *et al*., 2008) and docking simulations of the *E. coli* DmsD Tat proofreading chaperone with the Tat signal peptide of DmsA proposed that the peptide could adopt an extended conformation devoid of secondary structure when bound to DmsD (Stevens *et al*., 2009). However, this conclusion is based on an extrapolation of genetic and biochemical work onto a ligand-free chaperone structure, while the NapD/NapA work presented here demonstrates that amino acids involved in long-range indirect effects, including those very distant from the actually signal peptide binding site, are commonly identified by these methods. Overall, the comprehensive analysis of the NapD-NapAsp complex reported here shows that the twin-arginine signal peptide of NapA adopts an α-helical structure during NapD binding and clearly identifies the binding interface between the two molecules. Future work will focus on how the signal peptide is handed onwards from NapD to the Tat translocase to complete the transport of the nitrate reductase on the Tat pathway.

## Experimental procedures

### Bacterial strains and growth conditions

*Escherichia coli* strains SGQ061, SGQ101 and SGQ171 carry chromosomal mutations that produce variant NapA proteins bearing R6Q, K10Q, or A17Q substitutions respectively. The host strain used in the construction of these mutants was LCB2048 (Blasco *et al*., 1992), which lacks both respiratory nitrate reductases but retains NapA. Mutant alleles were first constructed on pMAK705 and then moved onto the chromosome by homologous recombination (Hamilton *et al*., 1989).

For *in vivo* Tat transport assays the (essentially ‘wild-type’) *E. coli* strain MG1655 was used. This strain was transformed with pUNI-NapA encoding a fusion between the NapA signal peptide and CAT as described by Maillard *et al*. (2007), or variants of that vector constructed by site-directed mutagenesis. Cultures were grown aerobically in Luria–Bertani (LB) medium supplemented with variable amounts of chloramphenicol and growth rates were monitored in a Biotek 2 instrument.

### Protein methods

NapD*^His^* was overproduced and purified as described by Maillard *et al*. (2007). Variations of ϕMalE-NapAsp*^His^* fusion protein were prepared by performing immobilized metal affinity chromatography (IMAC) under denaturating conditions using 20 mM Tris.HCl (pH 7.5), 250 mM NaCl, 2 mM DTT (Buffer A) containing 5 M urea. Bound proteins were refolded on the HisTrap HP column (GE Healthcare Bio-Sciences AB) by applying a gradient of 5-0 M urea in Buffer A over 18 column volumes. Refolded proteins were subsequently eluted by a linear gradient of 30 ml over 0-0.5 M imidazole in Buffer A. Proteins were dialysed and concentrated.

Isothermal titration calorimetry was performed at 28°C in a VP-ITC microcalorimeter (GE Heathcare). The syringe was charged with 100 µM NapD*^His^* (or variants) and the sample cell contained 1.4 ml of 10 µM of the appropriate MalE fusion protein. Titrations typically comprised of 35 × 8 µl injections of NapD*^His^*. Data analysis was performed with Origin software (GE Healthcare).

### Bacterial two hybrid system

Protein–protein interactions were detected *in vivo* using a bacterial two-hybrid system (Karimova *et al*., 1998). Interactions were estimated by measurement of β-galactosidase activity in the *E. coli* BTH101 reporter strain grown aerobically to mid-log phase at 30°C.

### NMR spectroscopy

Backbone and side-chain assignments of NapDAsp were obtained using the standard triple-resonance experiments HNCACB, CBCACONH, HN(CA)HA and (H)CCH-TOCSY experiments recorded on Varian *Inova* spectrometer operating at 600 MHz ^1^H resonance frequency. Three-dimensional ^15^N- and ^13^C-edited NOESY experiments were recorded on a Varian *Inova* spectrometer operating at 800 MHz ^1^H resonance frequency and equipped with a cold probe using an 100 ms mixing time. All data were processed using the NMRPipe program suite (Delaglio *et al*., 1995) and analysed using SPARKY. The program TALOS+ (Shen *et al*., 2009) was used for prediction of ϕ, ψ backbone angles and calculation of the S^2^–order parameter (Wishart *et al*., 1995). Per-residue chemical shift differences between NapD and the NapDAsp constructs were calculated using weighting factors (Ayed *et al*., 2001) 1.0, 0.154, 0.276 for ^1^H, ^15^N and ^13^C shifts respectively.

### Modelling of the NapDAsp complex

Using the first ensemble member of the structure of the free NapD as a starting point (PDB code 2jsx), residues 71–81 of NapD strand β4 were first adjusted and extended. Starting first with residues 71–74 and then repeating for each successive residue, its ϕ, Ψ backbone angles were set to the TALOS+ predicted values and NapD β-strand 4 was refined in a short simulated annealing procedure using the program YASARA (http://www.yasara.org) and the YASARA2 force field. A plausible β-strand extension was formed for NapD residues 75–78. Next, the structured residues N12-V25 of the NapA signal peptide were folded using the TALOS+ predicted ϕ, ψ backbone angles. The peptide was then positioned onto the NapD protein in such as fashion to generate the largest agreement with the observed chemical shift perturbation data. A random nature for NapA residues R5-A11 is not in accordance with the chemical shift perturbations observed for the strand β3-helix α2 region of the NapD molecule and would result in considerable steric clashes between the bulky NapD residue W5 and signal peptide residues R5, R6, F8 and M9. It is more likely that this region of the peptide is in intermediate exchange thus leading to line broadening beyond detection. NapA residues R5-A11 were therefore modelled as an ideal helix, which is in line with the conformation of the NapA signal peptide in the alternative 2pq4 NMR structure and our protein interaction data. To avoid steric clashes, the side-chain conformation of NapD residue Y76 and NapAsp residues F8 and M9 were changed. Preferred χ_1_, χ_2_ rotameric conformations, as derived from the WHATIF structural reference database, were tested and a selection was made on the basis of minimal steric overlap. Finally, the NapD-NapAsp complex was refined using the program YASARA and the YASARA2 force field in explicit solvent. The complete NapA signal peptide and the NapD side-chains of residues 5, 7, 9, 11, 33, 35, 36, 38, 43, 45, 47, 49, 75, 76 and 78, which together form the complete β-sheet interface, were allowed to adjust in this refinement. The structure of the resulting NapD-NapAsp complex together with its chemical shift data was then subjected to the CING validation server (http://nmr.cmbi.ru.nl/icing; results in Table S1).

### Site-directed and random mutagenesis and library screening

The plasmid pT25-NapD, from the bacterial two hybrid system, was used as a template to generate a library of random mutations in *napD* using error-prone PCR (error rate estimated at 1.0% and 1.5%). The method used was similar to that employed by Buchanan *et al*. (2008).

### *In vivo* nitrite production assays

To measure nitrate reductase activity the Grieß method of nitrite detection was used, essentially according to Maillard *et al*. (2007). In brief, cells were grown anaerobically in LB medium supplemented with 0.5% glycerol (v/v), 0.4% fumarate (w/v) and 0.2% nitrate (w/v). Nitrite concentration was measured by sampling 200 µl cell-free culture supernatant and adding 600 µl of 100 mM Tris.HCl (pH 7.6) and 400 µl of a 2:1 mixture of 4% sulphanilamide [in 25% (v/v) conc. HCl] and 0.08% (w/v) *N*-(1-naphthyl)ethylendiamine. Samples were incubated for 15 min at room temperature before the absorbance at 540 nm was recorded.
