# Supplementary material for: Overlapping transport and chaperone-binding functions within a bacterial twin-arginine signal peptide
Source: Mol Microbiol. 2012 Feb 27;83(6):1254–67. doi: 10.1111/j.1365-2958.2012.08005.x (PMC3712460; doi:10.1111/j.1365-2958.2012.08005.x)

Overlapping transport and chaperone-binding functions within a bacterial twin-arginine signal peptide.

Sabine Grahl, Julien Maillard, Chris AEM Spronk,
Geerten W Vuister and Frank Sargent

SUPPLEMENTARY INFORMATION

TABLE S1: Structural quality indicators as derived by the iCing structure validation server (http://nmr.cmbi.ru.nl/icing).

	2PQ4[1]	Model
**WHAT IF Z-scores**		
1st generation packing quality	0.3 +/- 0.6	0.60
2nd generation packing quality	3.0 +/- 1.5	-0.05
Ramachandran plot appearance	-5.5 +/- 0.2	-0.02
chi-1/chi-2 rotamer normality	-7.1 +/- 0.3	-2.26
Backbone conformation	-1.4 +/- 0.1	0.99
**WHAT IF RMS Z-scores**		
Bond lengths	1.237 +/- 0.001	1.16
Bond angles	0.865 +/- 0.001	0.56
Omega angle restraints	0.066 +/- 0.003	1.69
Side chain planarity	2.1 +/- 0.3	1.13
Improper dihedral distribution	1.516 +/- 0.003	0.96
Inside/Outside distribution	1.014 +/- 0.010	0.95
**PROCHECK NMR**		
core (%)	82.3	93.7
allowed (%)	16.7	5.3
generous (%)	1.0	1.1
disallowed (%)	0.0	0.0
**CING**		
Red (%)	40	20
Orange (%)	33	20
Green (%)	27	60

[1] Data accessible from the NRG-CING database: http://nmr.cmbi.ru.nl/NRG-CING/data/pq/2pq4/2pq4.cing/

FIG. S1: ^{1}H-^{15}N HSQC NMR spectra of NapD and NapDAsp. An overlay of ^{15}N-HSQC spectra of NapD in monomeric free state (*purple*) and the NapDAsp fusion protein (*green*). For points of reference the NapD E17 cross-peak, which is not affected upon NapAsp binding, and the NapD L10 crosspeak, which is dramatically shifted, are shown.

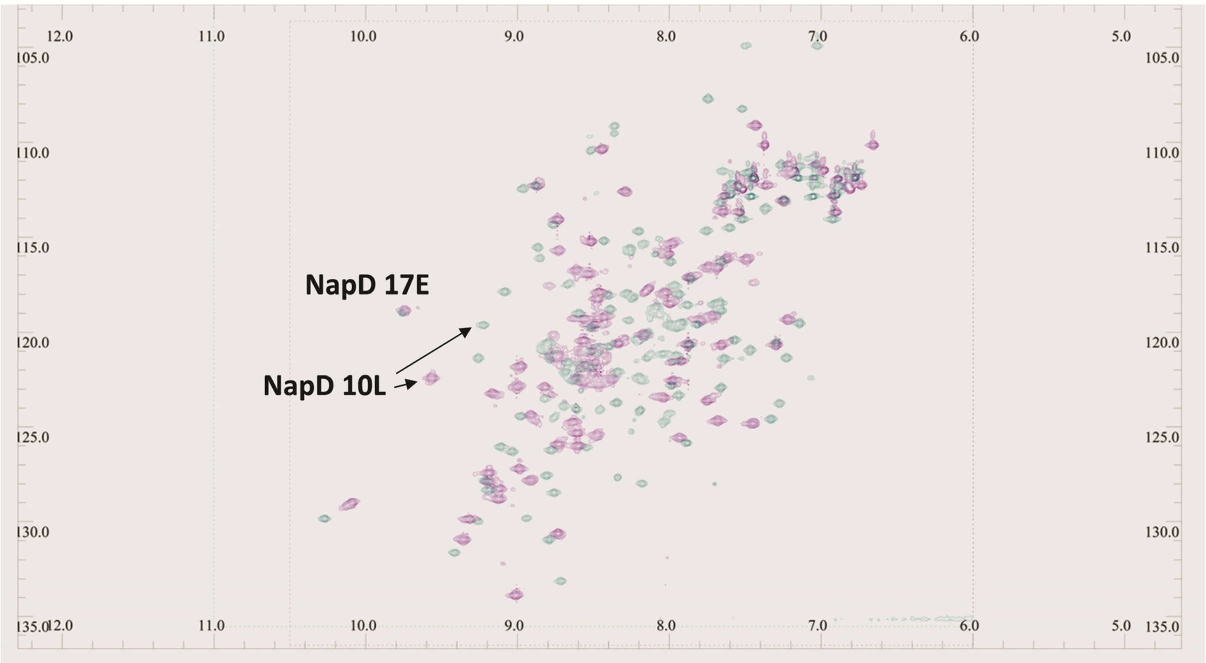

FIG. S2: ITC binding analysis of NapD suppressors and NapA A17Q signal peptide. All experiments were performed in Tris.HCl buffer under identical conditions and identical protein concentrations as described in materials and methods. Upper panels show raw data for heat effect during titration. Shown are ITC titrations of NapD suppressors A14T A71T **(A)**, A14T **(B)**, A71T **(C)**, I19F T59I **(D)**, I19F **(E)** and T59I **(F)** with MalE-NapA$_{SP}$ variant A17Q.

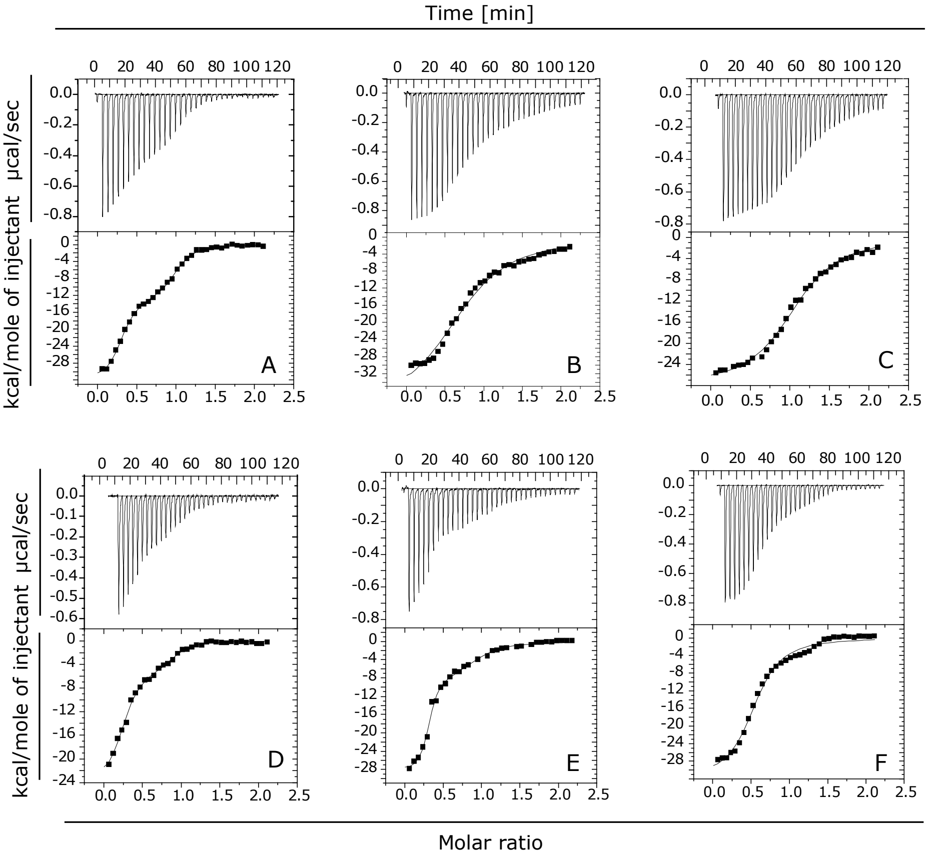

FIG. S3: Testing for enhanced Tat transport of NapA A17Q *in vivo* by the different NapD suppressor variants. *E. coli* strain SGQ171 (NapA A17Q) was transformed with plasmid pT25-NapD or a version expression the *napD* suppressor mutants. Nitrite production was measured *in vivo*. Strain SGQ171 and transformants are labelled as followed: SGQ171 (■), SGQ171/NapD (▲), SGQ171/NapD I19F (Δ), SGQ171/NapD A14T A71T (☐), SGQ171/NapD I19F T59I (○).

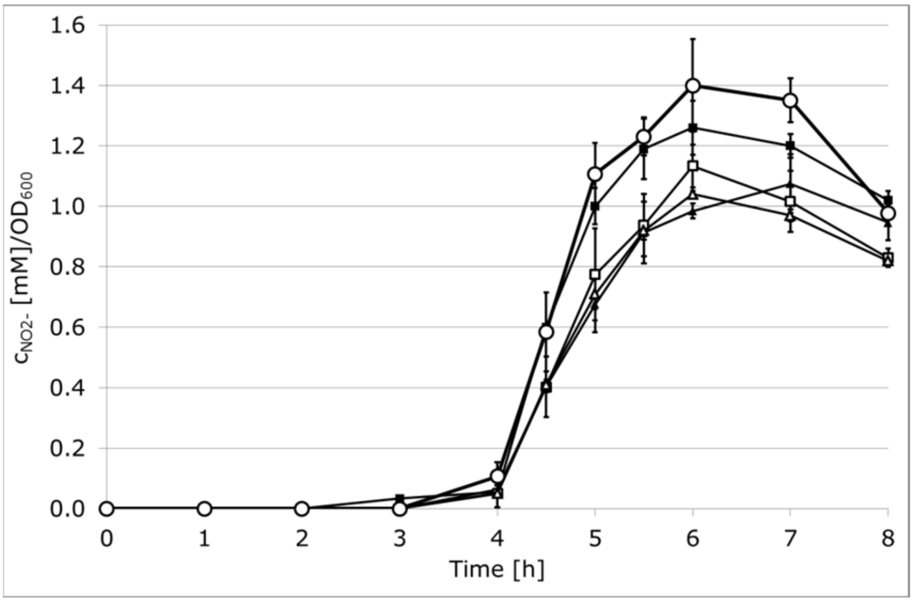

FIG. S4: Sequence alignment of different NapA twin-arginine signal peptides. Homologs from *Escherichia coli* (*Ec*), *Agrobacterium tumefaciens* (*At*), *Bradyrhizobium japonicum* (*Bj*), *Cupriavidus necator* (*Cn*), *Desulfovibrio desulfuricans* (*Dd*), *Paracoccus pantotrophus* (*Pp*), *Rhodobacter sphaeroides* (*Rs*) and *Shewanella oneidensis* (*So*) are shown. Identified residues for NapD binding by BTH and ITC are highlighted with an arrow. Highly conserved residues are underplayed in *orange*. Amino acid residues with similar properties are shown in *grey*.

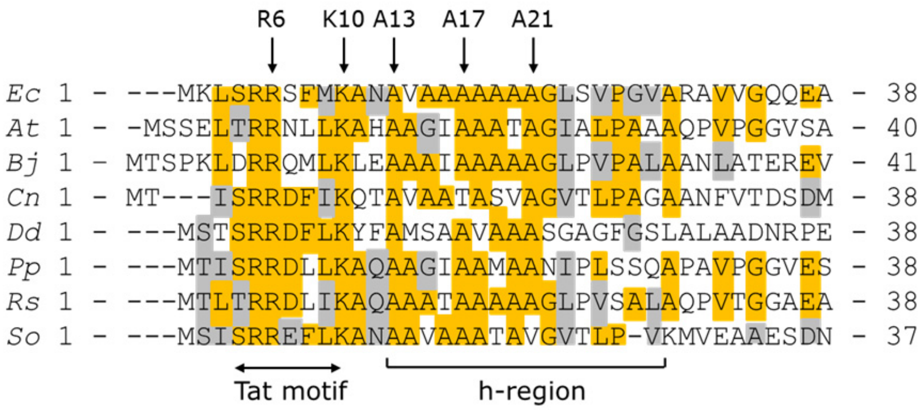

FIG. S5: Comparison of our NapDAsp complex with that of the alternative structure 2pq4.
(A) Superposition of 2pq4 (NapD in *blue* with NapA signal peptide in *cyan*) and our model presented in this work (NapD in *orange* with the NapA signal peptide in *yellow*). Note the differences in orientation of the helical NapA signal peptide. **(B)** Representation of 2pq4 in relation to the current experimental data presented in this work. NapD is shown in surface representation, colour-coded *blue* to *red* for increasing chemical shift perturbation effects observed in our experiments (Fig. 6 of the manuscript) and using *white* annotation for NapD residues. The NapA signal peptide is shown in ribbon and stick representation, using *yellow* colouring for crucial interacting residues and their annotation. Note that in the case of 2pq4 the position of the NapA signal peptide is not in agreement with our chemical shift data for the complex.

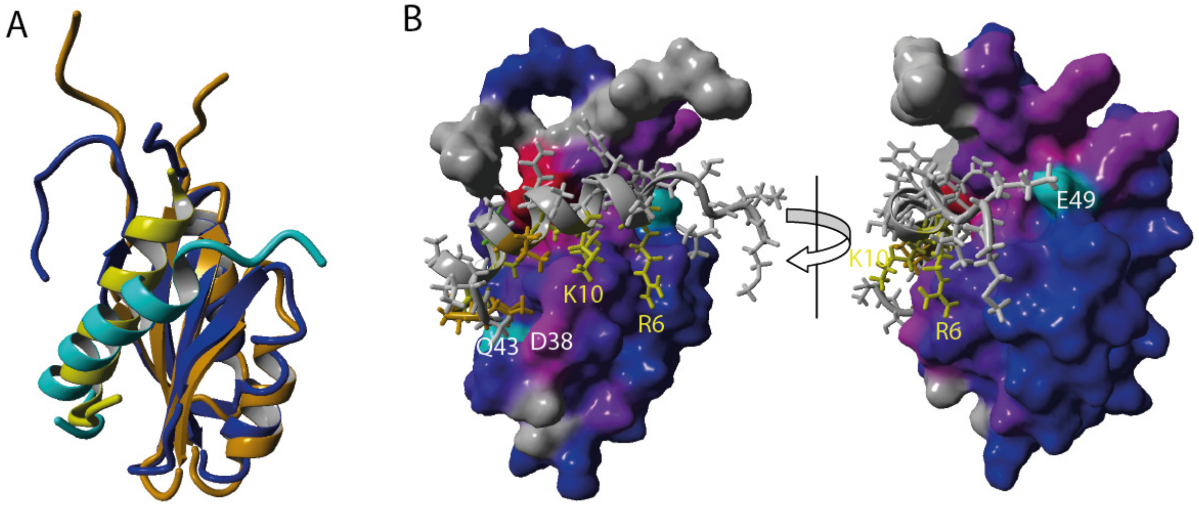

Supplement: Supplementary file 1 [file mmi0083-1254-SD1.pdf]
